# Altered function and maturation of primary cortical neurons from a 22q11.2 deletion mouse model of schizophrenia

**DOI:** 10.1038/s41398-018-0132-8

**Published:** 2018-04-18

**Authors:** Ziyi Sun, Damian J. Williams, Bin Xu, Joseph A. Gogos

**Affiliations:** 1 Department of Integrated Traditional Chinese and Western Medicine, State Key Laboratory of Biotherapy and Cancer Center, West China Hospital, Sichuan University, and Collaborative Innovation Center for Biotherapy, Chengdu, Sichuan 610041 China; 2 Department of Physiology and Cellular Biophysics, College of Physicians and Surgeons, Columbia University, New York, NY 10032 USA; 3 Columbia Stem Cell Core Facility, Department of Pathology and Cell Biology, Columbia University Medical Center, New York, NY 10032 USA; 4 Department of Psychiatry, Columbia University Medical Center, New York, NY 10032 USA; 5 Department of Neuroscience, Columbia University Medical Center, New York, NY 10032 USA

## Abstract

Given its high penetrance, clearly delineated and evolutionary conserved genomic structure, mouse models of the 22q11.2 deletion provide an ideal organism-based and cell-based model of this well-established disease mutation for schizophrenia. In this study we examined the development of changes in intrinsic properties, action potential firing and synaptic transmission using whole-cell patch-clamp recordings of cultured embryonic cortical neurons from *Df(16)A*^*+/−*^ and WT mice at DIV7 and DIV14, respectively. Compared to neurons from the WT littermates, significantly increased input resistance and decreased rising rate of action potential was observed in *Df(16)A*^*+/−*^ mice at DIV7 but not at DIV14 indicative of delayed neuronal maturation. Neurons from *Df(16)A*^*+/−*^ mice also showed significantly higher cellular excitability at both DIV7 and DIV14. Evaluation of Ca^2+^ homeostasis perturbation caused by 22q11.2 deletion using calcium imaging revealed a significantly lower amplitude of calcium elevation and a smaller area under the curve after depolarization in neurons from *Df(16)A*^*+/−*^ mice at both DIV7 and DIV14. Furthermore, the properties of inhibitory synaptic events were significantly altered in *Df(16)A*^*+/−*^ mice. We identified changes in mRNA expression profiles, especially in ion channels, receptors, and transporters that may underlie the neurophysiological effects of this mutation. Overall, we show a number of alterations in electrophysiological and calcium homeostatic properties of embryonic cortical neurons from a 22q11.2 deletion mouse model at different culture times and provide valuable insights towards revealing disease mechanisms and discovery of new therapeutic compounds.

## Introduction

While much progress has been made recently in understanding the genetic causes of psychiatric illnesses, there remain many unresolved questions pertaining to the underlying neural mechanisms. 22q11.2 deletions represent one of the greatest known genetic risk factors for schizophrenia (SCZ). De novo 22q11.2 deletions account for up to 1–2% of sporadic SCZ cases^1–3^ and increase the chance of developing SCZ by ~30-fold. Most affected individuals carry a 3-Mb hemizygous deletion, whereas 7% have a nested 1.5-Mb deletion spanning 28 known genes^4–6^.

A mouse model (*Df(16)A*^*+/−*^)^7^, carrying an engineered orthologous deletion on mouse chromosome 16 encompassing all but one of the genes encoded in the 1.5-Mb region, is a particularly powerful tool for deciphering how this genetic lesion increases risk for SCZ. Previous electrophysiological analysis of the *Df(16)A*^*+/*−^ mice was performed on various preparations, including autaptic cultures of hippocampal neurons^8^, slice preparations from hippocampus^9^, or prefrontal cortex^10–13^ as well as from live behaving mice^11,14,15^. Although some differences were observed related to the brain area tested, the mode of stimulation (electrical vs. optogenetic) and the age of the mice tested, taken together, previous studies on this mouse model revealed robust alterations of electrophysiological properties, synaptic function and plasticity, dopaminergic and GABAergic neuromodulation as well as structural and functional neuronal connectivity both within and between brain areas, particularly in hippocampus and prefrontal cortex^9–11,13,14,16,17^. These effects are in part due to chromosomal deficiency induced disruptions in microRNA processing and protein palmitoylation^7,8,18^, although the impact of these and other processes affected by this genomic lesion, on the membrane properties, ionic currents, and synaptic physiology of mutant embryonic cortical neurons remains largely unknown.

Using the *Df(16)A*^*+/−*^ mouse strain we investigated the physiological properties of cultured embryonic cortical neurons from mutant and wild-type (WT) mice at two different time points (DIV7 and DIV14) and explored how the deletion affects the membrane properties, firing patterns, and synaptic activity during neural development and maturation in vitro. We provide evidence that this mutation in mouse resembling a SCZ risk allele results in alterations in a number of properties of cultured embryonic cortical neurons, indicative of delayed maturation, altered synaptic activities, hyper excitability as well as perturbation of intracellular calcium homeostasis of mutant neurons. Comparison of mRNA expression profiles implicated transcriptional alterations of ion channels, receptors, and transporters caused by the 22q11.2 deletion that may ultimately affect the membrane and synaptic properties, cell excitability, and calcium homeostasis.

## Methods

### Cell culture

Mouse cortical astrocytes were isolated from P0 WT mouse pups using methods described previously^19^. Nearly pure astrocytes were plated at a density of 5 × 10^4^ cells per 35 mm dish. The astrocytes were maintained 4 to 5 days to reach confluency in the dish and formed a monolayer astrocyte bed. Primary embryonic cortical neurons were isolated from E17.5 mouse embryos which were generated by crossing male *Df(16)A*^*+/−*^ mice with female C57 mice. Cortices were dissected bilaterally and digested with 0.25% trypsin at 37 °C for 30 min, then dissociated using fire-polished Pasteur pipettes. Neurons were plated at a density of 1 × 10^5^ cells per 35 mm dish, which has astrocyte bed pre-plated. Neurons were initially maintained in modified DMEM media for 3 h, which then were replaced with neurobasal media supplemented with B27 and Glutamax (Invitrogen, USA) in which the neurons were cultured for 7 (DIV7) to 14 (DIV14) days depending on the experiments.

### Electrophysiology

Electrophysiological signals were acquired using Multiclamp 700B amplifier, 1332A DigiData and pClamp10 software (Lowpass filter frequency: 10,000 Hz) (Molecular Devices, USA). Whole-cell patch-clamp recordings were performed using borosilicate glass pipettes (initial resistance 3.0–6.0 MΩ). An external solution was used that contained (in mM): NaCl 145, KCl 5, HEPES 10, Glucose 10, CaCl_2_ 2, MgCl_2_ 2, pH 7.3 with NaOH, adjusted to 325 mOsm with sucrose. An internal solution was used that contained (in mM): KMeSO_4_ 130, NaMeSO_4_ 10, EGTA 10, CaCl_2_ 1, HEPES 10, MgATP 5, Na_2_GTP 0.5, pH 7.2 with KOH, adjusted to 290 mOsm with sucrose. Input resistance was measured in the voltage-clamp mode at −70 mV from the current response to a 5 mV hyperpolarizing voltage step and calculated by Ohm’s law from the current change. Transient sodium channel currents are reported as inward peak currents during a series of voltage steps from −60 mV to +60 mV. Sustained potassium channel currents are reported as the quasi steady state currents at the end of a 100 ms voltage step. For assessing neuronal excitability, action potential (AP) firing was recorded in current-clamp mode in response to incremental, depolarizing current injections of 500 ms duration (10 pA increment of 25 steps). The number of AP firings was plotted to the corresponding current steps. Waveform analysis of initial AP firing was performed using standard Clampfit routines as follows: “Half width”, the AP width at the half height; “Rising rate”, the slope of the rising event from 10 to 90% of the maximum; “Decay rate”, the slope of the decay event from 90 to 10% of the maximum. The rheobase was measured by a series of short (5 ms duration) current injections (20 pA increment of 80 steps) and calculated as the minimal current amplitude to elicit the initial AP. In current-clamp mode, the resting membrane potential (RMP) of all cells was adjusted to −70 mV by injection of a small standing current. Spontaneous excitatory postsynaptic currents (sEPSCs) and spontaneous inhibitory postsynaptic currents (sIPSCs) were recorded in voltage-clamp mode, holding cells at −60 and 0 mV, respectively. Miniature excitatory postsynaptic currents (mEPSCs) and miniature inhibitory postsynaptic currents (mIPSCs) were recorded using the same voltage protocol as sEPSCs and sIPSCs, respectively. The Cs^+^ based internal solution was used for miniature synaptic event recordings (in mM): CsMeSO_4_ 110, NaMeSO_4_ 10, EGTA 10, CaCl_2_ 1, HEPES 10, TEA 10, QX-314 5, MgATP 5, Na_2_GTP 0.5, pH 7.2 with KOH, adjusted to 290 mOsm with sucrose, and 1 µM tetrodotoxin (TTX) was present in the external solution to block the spontaneous AP firing. Synaptic events were sampled for 5 min and were analyzed using MiniAnalysis 6.0, with which the amplitude and frequency of synaptic events were calculated. Statistical analyses were performed using SigmaPlot 9.0 and GraphPad Prism 4. Data are presented as means ± SEM.

### Calcium imaging and analysis

Cover slips containing neurons were incubated with 5 µM Fura-2 AM, ratio-metric calcium indicator dye (Life Sciences, USA) for 30 min at room temperature. After washing, cover slips were mounted on a Nikon Eclipse TE 3500 inverted microscope equipped with a 40 × 1.30 NA objective (Nikon, USA), a pco.EDGE CMOS camera (pco, Germany), a Lambda LS light source, a Lambda LS-2 filter wheel with 340 and 380 nm excitation filters (both Sutter, USA). Images at each excitation wavelength were acquired at 1 Hz. Cells were perfused by Ringer’s solution at a rate of 1 ml/min. Brief applications (2, 5, and 10 s) of high concentration KCl (30 mM) solution were carried out using a custom built focal application system located ~100 µm from the field of view. Measurements were obtained from two fields of cells on each cover slip. Images were acquired for 60 s with 6 s control period followed by 54 s after application of KCl. Following acquisition, the 340/380 ratio of each pair of images was calculated on a pixel by pixel basis using FIJI software v. 1.4 (www.fiji.sc). Regions of interests were drawn manually using the morphology of the cells from a DIC image as a template. Only neurons with a peak value 20% higher than the baseline level and recovered 90% more than of the peak value were included in the analysis. Further quantification was carried out using Igor Pro v. 6 (Wavemetrics, USA) and R v. 3 (www.R-project.org).

### Total RNA isolation and RNA sequencing

Ten cortical neuron cultures (1.2 × 10^6^ for each sample) were collected from 5 WT mice and 5 *Df16(A)*^*+/−*^ mice at DIV7, all E17.5 male embryos. Total RNA was isolated from the cortical neurons using miRNeasy kit (Qiagen, USA) according to the instructions of the manufacturer. RNA was suspended in RNase-free water. The concentration and purity of each sample was determined by spectrophotometer (ND1000; Nanodrop, USA) and confirmed by Microchip Gel Electrophoresis (Agilent, USA) using Agilent 2100 Bioanalyzer Chip according to the instructions of the manufactures. A poly-A pull-down step was performed to enrich mRNAs from total RNA samples (200 ng to 1 µg per sample, RIN > 8 required) and proceeded on library preparation by using Illumina TruSeq RNA prep kit. Libraries were then sequenced using Illumina HiSeq2000 at Columbia Genome Center. Multiplex samples wit unique barcodes were mixed in each lane, which yields targeted number of single-end 100 bp reads for each sample, as a fraction of 180 million reads for the whole lane. RTA software (Illumina, USA) was used for base calling and bcl2fastq (version 1.8.4) for converting BCL to fastq format, coupled with adaptor trimming. The reads were mapped to a reference genome (Mouse: UCSC/mm9) using Tophat^20^ (version 2.0.4) with four mismatches (--read-mismatches = 4) and 10 maximum multiple hits (--max-multihits = 10).

### Differential expression analysis

DESeq2, an R package based on a negative binomial distribution that models the number reads from RNA-seq experiments and test for differential expression, was used to determine differentially expressed genes (DEGs) between mutants and control samples^21^. The list of significantly DEGs was defined at false discovery rate (FDR) adjusted *p*_adj_ < 0.05.

### Gene ontology and gene-set enrichment analysis

To determine a common functional relationship among the top DEGs, the enrichment of biological processes was tested using Toppgene for gene ontology (GO) analysis with default settings. The GAGE software^22^ was also used to conduct a gene set enrichment analysis to analyze genes related to synaptic structure and functions based on the KEGG pathway database (http://www.genome.jp/kegg/pathway.html).

## Results

### *Df(16)A*^*+/−*^ embryonic cortical neurons show altered passive membrane properties at early culture time points

**Fig. 1:**
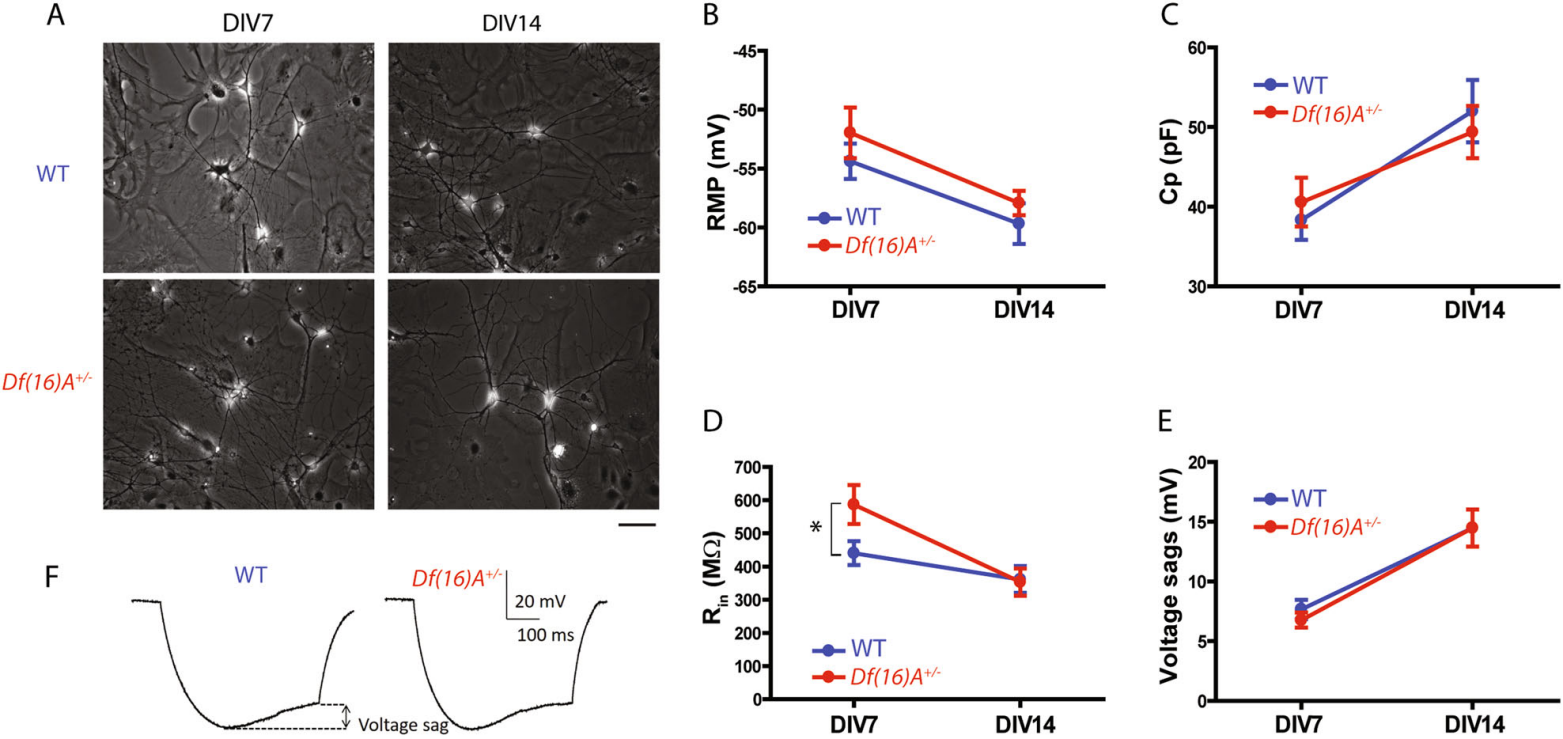
Passive membrane properties of WT and *Df(16)A*^*+/−*^ cortical neurons. **a** Bright field images of WT and *Df(16)A*^*+/−*^ cortical neurons co-cultured with astrocytes at DIV7 and DIV14. Scale bar = 50 µm. **b** Quantitative data of resting membrane potential (RMP) measured in WT and *Df(16)A*^*+/−*^ cortical neurons at DIV7 and DIV14, respectively. There was no significant difference between genotypes at both time points. **c** Quantitative data of cell capacitance (*C*_p_) in WT and *Df(16)A*^*+/−*^ cortical neurons at DIV7 and DIV14, respectively. There was no significant difference between genotypes at both time points. **d** Quantitative data of input resistance (*R*_in_) in WT and *Df(16)A*^*+/−*^ cortical neurons at DIV7 and DIV14, respectively. *Df(16)A*^*+/−*^ cortical neurons showed significant higher *R*_in_ than that of WT neurons at DIV7 (*t* test, *p < 0.05). **e** Quantitative data of voltage sags caused by HCN-mediated currents in WT and *Df(16)A*^*+/−*^ cortical neurons at DIV7 and DIV14, respectively. There was no significant difference between genotypes at both time points. **f** Sample traces of HCN-caused voltage sags in WT and *Df(16)A*^*+/−*^ cortical neurons at DIV7. In the presence of HCN-mediated currents, a negative current step (hyperpolarizing the cell membrane to −150 mV) induced a depolarizing voltage sag (arrows, left panel)

Isolated primary embryonic cortical neurons were co-cultured with monolayer astrocytes for 7 to 14 days in vitro and analyzed at two time points. Under the structural and metabolic support of astrocytes, cell cultures were maintained healthy for more than 2 weeks. Neurons with typical pyramidal cell morphology (bright pyramid-shaped cell bodies and multiple extended processes, Fig. 1a) were selected for recording. The identity of these neurons was independently verified by immunostaining with pyramidal cell/neuron-specific markers such as EMX1, Tuj1, MAP2, and Neu N (Fig. S1A). To analyze the maturation process and to compare the intrinsic properties between genotypes, the passive membrane properties were characterized using whole-cell patch-clamp recordings at two time points: DIV7 and DIV14.

The RMP was determined mainly by non-gated ion channels and a hyperpolarized RMP is considered a universal feature of mature neurons. RMP of WT neurons was –54.38 ± 1.50 mV (*n* = 29) at DIV7, and was significantly shifted to –59.67 ± 1.72 mV (*n* = 27) at DIV14 (*t* test, *p* < 0.05, Fig. 1b). Similarly, a significant change of RMP was observed in *Df(16)A*^*+/−*^ cortical neurons from –51.96 ± 2.16 mV (*n* = 28) at DIV7 to –57.92 ± 1.04 mV (*n* = 26) at DIV14 (*t* test, *p* < 0.05, Fig. 1b). Both WT and *Df(16)A*^*+/−*^ neurons showed a significantly negative shift of RMP from DIV7 to DIV14, indicative of a maturation process during neuronal development. However, there was no difference of RMP between genotypes at either time point (*t* test, *p* = 0.369 at DIV7; *p* = 0.397 at DIV14).

Cell capacitance (*C*_p_) scales with the cell surface area, which is often used as a stand-in for a measurement of cell size^23^. Capacitance of WT neurons increased significantly from 38.30 ± 2.47 pF at DIV7 (*n* = 29) to 52.00 ± 3.91 pF (*n* = 27) at DIV14 (*t* test, *p* < 0.05, Fig. 1c). In contrast, *Df(16)A*^*+/−*^ neurons showed a barely significant increase in cell capacitance from 40.57 ± 3.07 mV (*n* = 28) at DIV7 to 49.38 ± 3.28 mV (*n* = 26) at DIV14 (*t* test, *p* = 0.055, Fig. 1c). These results indicate an increase in cell size of both genotypes during culture. No significant genotypic differences were observed for *C*_p_ at either time point (*t* test, *p* = 0.566 at DIV7 and *p* = 0.610 at DIV14), consistent with measurements of cell body area, which showed no difference between genotypes at either time point (Fig. S1B, *t* test, *p* = 0.185 at DIV7 and *p* = 0.637 at DIV14).

Input resistance (*R*_in_) depends on the density of open channels at RMP and the cell size. A decreased *R*_in_ suggests increased ion channel activities at the cell membrane. *Df(16)A*^*+/−*^ neurons showed a significant decrease in *R*_in_ from DIV7 to DIV14 (*t* test, *p* < 0.05), while WT neurons did not (*t* test, *p* = 0.146). Compared to WT neurons (440.38 ± 35.83 MΩ, *n* = 29), *Df(16)A*^*+/−*^ neurons exhibited a significant higher *R*_in_ (586.75 ± 58.66 MΩ, *n* = 28) at DIV7 (*t* test, *p* < 0.05), but a comparable *R*_in_ at DIV14 (WT, 361.03 ± 40.45 MΩ, *n* = 27 and *Df(16)A*^*+/−*^, 353.03 ± 41.16 MΩ, *n* = 26; *t* test, *p* = 0.890, Fig. 1d). Since the cell size and *C*_p_ are comparable between genotypes at DIV7, lower conductance through hyperpolarization-activated cyclic nucleotide-gated (HCN) channels or potassium leak channels would be likely mechanisms underlying the higher *R*_in_ observed in *Df(16)A*^*+/−*^ neurons at DIV7.

HCN-mediated current (*I*_h_) was measured from the amplitude of the voltage “sag”, a characteristic delayed depolarization caused by the slow activation of HCN channels^24^, as shown in Fig. 1f. To ensure that the *I*_h_ can be compared between genotypes, the amplitude of the hyperpolarizing current injection was adjusted to elicit a membrane potential of −150 mV at the start of the step. There was no significant difference of voltage sags between genotypes at either DIV7 (WT, 7.65 ± 1.27 mV, *n* = 20, and *Df(16)A*^*+/−*^ 6.76 ± 0.97 mV, *n* = 17, *t* test, *p* = 0.620) or DIV14 (WT, 14.47 ± 2.25 mV, *n* = 19, and *Df(16)A*^*+/−*^, 11.17 ± 1.87 mV, *n* = 18, *t* test, *p* = 0.135) (Fig. 1e). These results suggest that the higher *R*_in_ in *Df(16)A*^*+/−*^ neurons at DIV7 was not caused by the HCN channels, and therefore, may be more dependent on potassium leak channels (Discussion section).

### *Df(16)A*^*+/−*^ embryonic cortical neurons show altered action potential properties at early culture time points

**Fig. 2:**
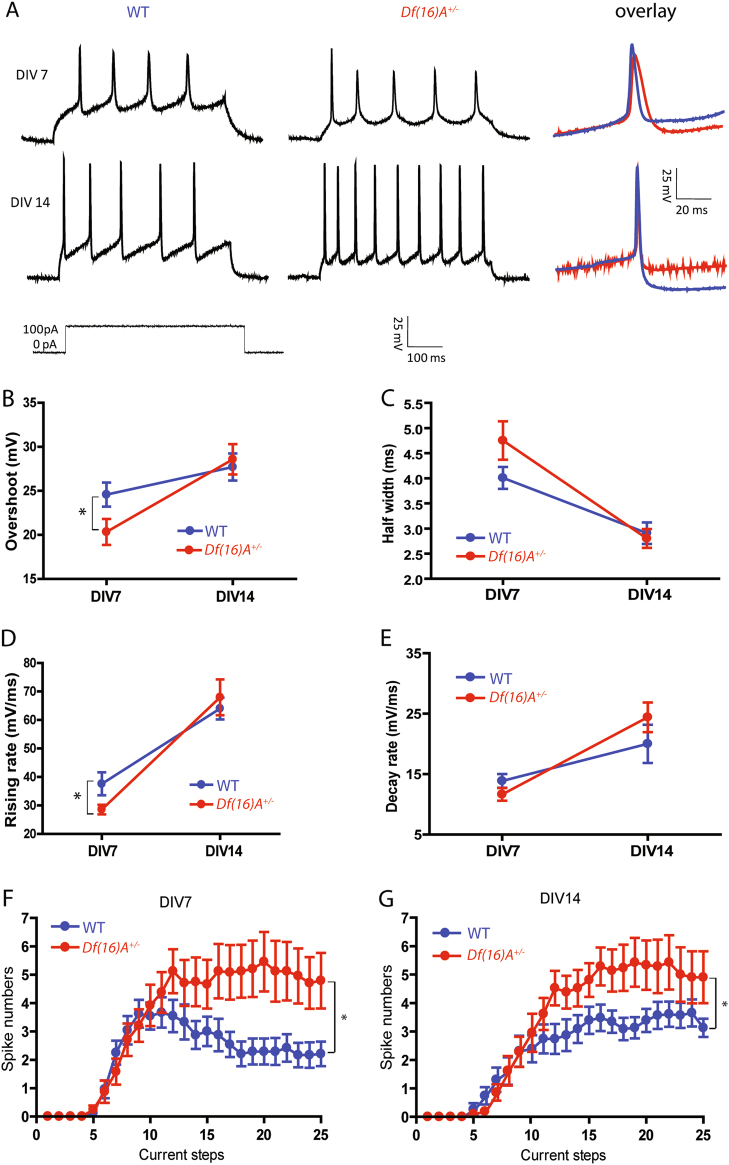
Action potential properties and cell excitability of WT and *Df(16)A*^*+/−*^ cortical neurons. **a** Representative action potential trains in WT (left panel) and *Df(16)A*^+/−^ neurons (middle panel) at DIV7 (upper panel) and DIV14 (lower panel), in response to a 500 ms depolarizing 100 pA current step from −60 mV. A close up of the WT (blue trace) vs. *Df(16)A*^+/−^ neuron (red trace) action potential waveforms overlaid (right panel) at both DIV7 and DIV14. **b** Quantitative data of overshoot (AP amplitude) in WT and *Df(16)A*^*+/−*^ cortical neurons at DIV7 and DIV14, respectively. *Df(16)A*^*+/−*^ cortical neurons showed significant decrease in overshoot at DIV7 compared to WT neurons (*t* test, *p < 0.05). **c** Quantitative data of half width (AP duration) in WT and *Df(16)A*^*+/−*^ cortical neurons at DIV7 and DIV14, respectively. There was no significant difference between genotypes at both time points. (**d**) Quantitative data of rising rate in WT and *Df(16)A*^*+/−*^ cortical neurons at DIV7 and DIV14, respectively. *Df(16)A*^*+/−*^ cortical neurons showed significant decrease in rising rate at DIV7 compared to WT neurons (*t* test, * *p* < 0.05). **e** Quantitative data of decay rate in WT and *Df(16)A*^*+/−*^ cortical neurons at DIV7 and DIV14, respectively. There was no significant difference between genotypes at both time points. **f** At DIV7, *Df(16)A*^+/−^ neurons (red) fired more action potentials than did WT neurons (blue) in response to large current injections for 500 ms when resting membrane potential was adjusted to −60 mV (2-way repeated measures ANOVA, genotype × current: *p < 0.05. Bonferroni post tests showed significant genotypic differences at current injections of 150, 160, 170, 180 and 200 pA). **g** At DIV14, *Df(16)A*^+/−^ neurons (red) fired more action potentials compared to WT neurons (blue) in response to large current injections for 500 ms when resting membrane potential was adjusted to −60 mV (2-way repeated measures ANOVA, genotype × current: *p < 0.05. Bonferroni post tests showed no significant difference at single current steps)

We characterized AP firing using current-clamp recordings. Repetitive AP firings were induced in both WT and *Df(16)A*^*+/−*^ neurons as early as DIV7, and more mature APs were induced at DIV14 (Sample traces are shown in Fig. 2a). To evaluate the maturation of APs and to compare the properties of APs between genotypes, we measured the amplitude (overshoot) and duration (half width) of first initiated AP. An increased amplitude (Fig. 2b) and a decreased duration (Fig. 2c) of AP were observed in both WT and *Df(16)A*^*+/−*^neurons from DIV7 to DIV14, indicating a maturation process of AP in both genotypes over time in culture.

Compared to WT neurons (24.58 ± 1.34 mV, *n* = 27), *Df(16)A*^*+/−*^ neurons showed significant lower AP amplitudes (20.34 ± 1.42 mV, *n* = 25; *p* < 0.05, *t* test, Fig. 2b) at DIV7 but comparable AP amplitudes at DIV14 (WT, 27.71 ± 1.53 mV, *n* = 24 and *Df(16)A*^*+/−*^, 28.58 ± 1.72 mV, *n* = 23, *p* = 0.706, *t* test, Fig. 2b). *Df(16)A*^*+/−*^ neurons showed more broadened AP duration at DIV7, but the difference was not significant (WT, 4.00 ± 0.21 ms, and *Df(16)A*^*+/−*^ 4.75 ± 0.37 ms, *p* = 0.091, *t* test, Fig. 2c), and there was no difference of AP duration at DIV14 either (WT, 2.91 ± 0.21 ms and *Df(16)A*^*+/−*^, 2.80 ± 0.19 ms, *p* = 0.710, *t* test, Fig. 2c).

The kinetic process of APs was then assessed by measuring their rising and decay rate. Compared to WT neurons (37.59 ± 3.97 mV/ms, *n* = 27), *Df(16)A*^*+/−*^ neurons displayed a significantly lower rising rate (28.56 ± 1.60 mV/ms, *n* = 25, *p* < 0.05, *t* test, Fig. 2d) at DIV7, but a comparable rising rate between genotypes at DIV14 (WT, 64.01 ± 3.81 mV/ms, *n* = 24; *Df(16)A*^*+/−*^ 67.96 ± 6.30 mV/ms, *n* = 23, *p* = 0.590, *t* test, Fig. 2d). There was no genotypic difference in decay rates at either DIV7 (WT, 13.87 ± 1.13 mV/ms, *n* = 27 and *Df(16)A*^*+/−*^, 11.64 ± 1.01 mV/ms, *n* = 25, *p* = 0.159, *t* test, Fig. 2e) or DIV14 (WT, 20.01 ± 3.17 mV/ms, *n* = 24 and *Df(16)A*^*+/−*^, 24.41 ± 2.47 mV/ms, *n* = 23, *p* = 0.282, *t* test, Fig. 2e). Moreover, the rheobases of APs were comparable between genotypes at DIV7 (WT, 894.48 ± 34.37 pA, *n* = 27 and *Df(16)A*^*+/−*^, 833.21 ± 26.60 pA, *n* = 25, *p* = 0.156, *t* test, Fig. S1C) and DIV14 (WT, 864.07 ± 40.18 pA, *n* = 24 and *Df(16)A*^*+/−*^, 850.00 ± 42.93 pA, *n* = 23, *p* = 0.811, *t* test, Fig. S1C). Finally, there was no significant difference of tau values between genotypes at either DIV7 (*Df(16)A*^*+/−*^ neurons: 46.22 ± 5.67 ms, *n* = 25 and WT neurons: 40.75 ± 4.25 ms, *n* = 27 *p* = 0.262, *t* test, Fig. S1D) or DIV14 (*Df(16)A*^*+/−*^ neurons: 31.85 ± 2.89 ms, *n* = 23 and WT neurons: 34.56 ± 2.72 ms, *n* = 24, *p* = 0.502, *t* test, Fig. S1D).

To investigate potential mechanisms underlying altered AP properties in *Df(16)A*^*+/−*^ neurons, we assessed Na^+^ and K^+^ currents in these neurons, which are critical for the AP generation in nerve cells. Large transient inward currents (which can be completely blocked by 1 µM TTX, data not shown) and sustained outward currents were reliably induced in response to the voltage steps (sample trace and enlargement are shown in Fig. S1E), which are commonly used to estimate the magnitude of transient Na^+^ (*I*_Na_) and sustained K^+^ (*I*_K_) currents. The *I*–*V* curves revealed that *I*_Na_ (Fig. S1F) progressively increased in WT and *Df(16)A*^*+/−*^ neurons from DIV7 to DIV14, paralleling the maturation of APs. At DIV7, *I*_Na_ showed a reduction in *Df(16)A*^*+/−*^ neurons (−1386.91 ± 176.84 pA, *n* = 13) compared to WT neurons (−1828.31 ± 270.53 pA, *n* = 12, *p* < 0.05, Fig. S1F) at −30 mV, which may partly contribute to the decreased amplitude and slower rising rate of APs in *Df(16)A*^*+/−*^ neurons at this time point. However, overall there was no genotypic difference for *I*_Na_ at either DIV7 or DIV14 (Fig. S1F, *p* = 0.425 at DIV7 and *p* = 0.656 at DIV14, 2-way RM ANOVA). Furthermore, there was no genotypic difference for *I*_K_ observed at either DIV7 or DIV14 (Fig. S1G, *p* = 0.125 at DIV7 and *p* = 0.213 at DIV14, 2-way RM ANOVA).

### *Df(16)A*^*+/−*^ embryonic cortical neurons show enhanced cell excitability

Cell excitability was further assessed by performing a set of depolarizing current pulses (500 ms duration with 10 pA increment of 25 sweeps). At DIV7, *Df(16)A*^*+/−*^ neurons (*n* = 24) fired significantly more APs than WT neurons (*n* = 24) over the whole range of current steps (Fig. 2f, *p* < 0.05, 2-way RM ANOVA). Bonferroni post tests showed significant genotypic differences at current injections of 150,160,170,180, and 200 pA. Consistently, *Df(16)A*^*+/−*^ neurons (*n* = 21) fired significantly more APs overall than WT neurons (*n* = 23) at DIV14 (Fig. 2g, *p* < 0.05, 2-way RM ANOVA). Bonferroni post tests showed no significant genotypic difference at each single step. The increased AP firing rate in *Df(16)A*^*+/−*^ neurons indicated an enhanced cell excitability at both DIV7 and DIV14. Since the input resistance differs between WT and *Df(16)A*^*+/−*^ neurons at DIV7, the observed excitability difference at DIV7 is likely due to differences in passive membrane properties.

### *Df(16)A*^*+/−*^ embryonic cortical neurons show altered inhibitory synaptic activity

**Fig. 3:**
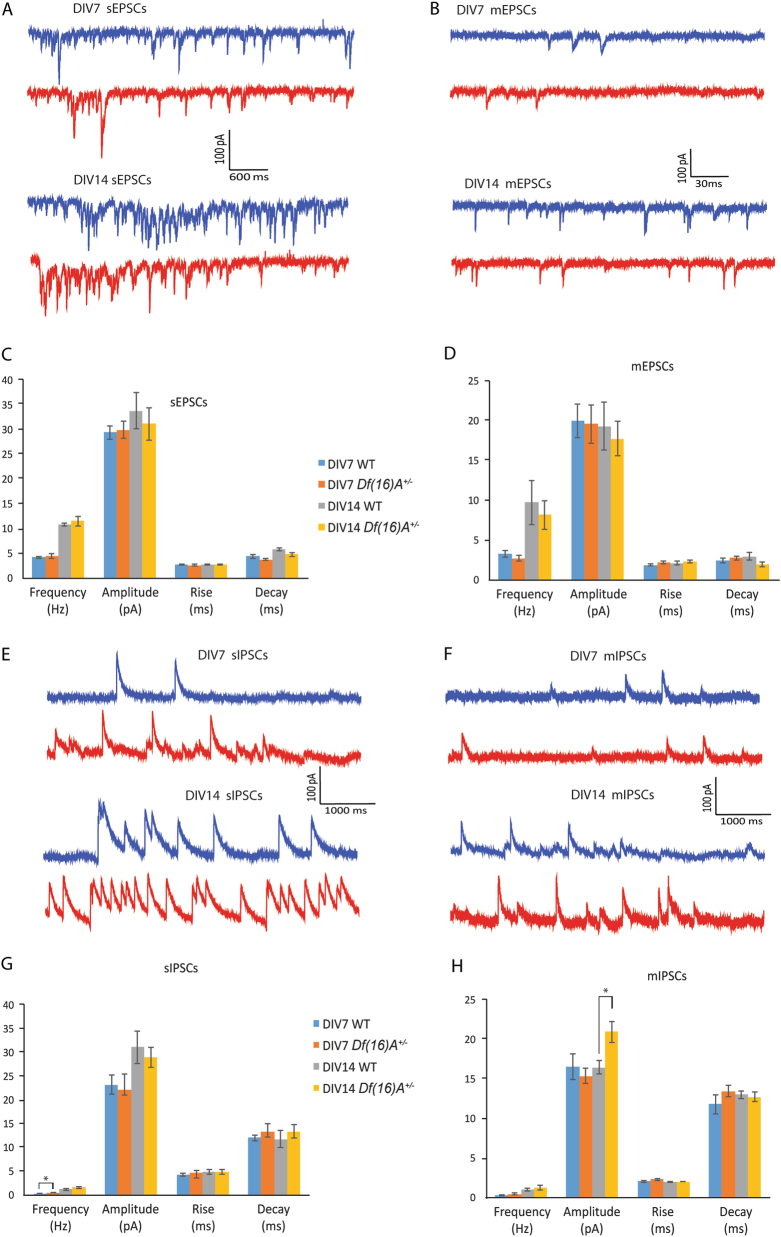
Synaptic properties of WT and *Df(16)A*^*+/−*^ cortical neurons. **a** Sample traces of sEPSCs from WT (blue trace) and *Df(16)A*^*+/−*^ (red trace) neurons at DIV7 (upper panel) and DIV14 (lower panel), respectively. Scale bar as shown in inset. **b** Sample traces of mEPSCs from WT (blue trace) and *Df(16)A*^*+/−*^ (red trace) neurons at DIV7 (upper panel) and DIV14 (lower panel), respectively. Scale bar as shown in inset. **c** Bar graphs of sEPSCs of WT and *Df(16)A*^*+/−*^ cortical neurons at DIV7 and DIV14. No significant difference was observed between genotypes. **d** Bar graphs of mEPSCs of WT and *Df(16)A*^*+/−*^ cortical neurons at DIV7 and DIV14. No significant difference was observed between genotypes. **e** Sample traces of sIPSCs from WT (blue trace) and *Df(16)A*^*+/−*^ (red trace) neurons at DIV7 (upper panel) and DIV14 (lower panel), respectively. Scale bar as shown in inset. **f** Sample traces of mIPSCs from WT (blue trace) and *Df(16)A*^*+/−*^ (red trace) neurons at DIV7 (upper panel) and DIV14 (lower panel), respectively. Scale bar as shown in inset. **g** Bar graphs of sIPSCs of WT and *Df(16)A*^*+/−*^ cortical neurons at DIV7 and DIV14. *Df(16)A*^*+/−*^ cortical neurons showed significant increase in frequency at DIV7 compared to WT neurons (*t* test, *p < 0.05). **h** Bar graphs of mIPSCs of WT and *Df(16)A*^*+/−*^ cortical neurons at DIV7 and DIV14. *Df(16)A*^*+/−*^ cortical neurons showed significant increase in amplitude at DIV14 compared to WT neurons (*t* test, *p < 0.05)

To explore whether *Df(16)A*^*+/−*^ affects synaptic function, sEPSCs were first investigated in WT and *Df(16)A*^*+/−*^ neurons. Sample traces of sEPSCs are shown in Fig. 3a. Overall the frequency and amplitude of sEPSCs were comparable between WT and *Df(16)A*^*+/−*^ neurons at both DIV7 and DIV14 (Fig. 3c and Table S1A). There were no genotypic differences in rising time or decay time at both time points either (Fig. 3c and Table S1A).

mEPSCs were further recorded in the presence of 1 µM TTX. Sample traces of mEPSCs are shown in Fig. 3b. Overall the frequency and amplitude of mEPSCs were also comparable between WT and *Df(16)A*^*+/−*^ neurons at both DIV7 and DIV14 (Fig. 3d and Table S1B). There was no genotypic difference in rising time or decay time at both DIV7 and DIV14 (Fig. 3d and Table S1B).

sIPSCs were recorded at a holding potential of 0 mV. Sample traces of sIPSCs are shown in Fig. 3e. At DIV7, there was a significant increase in the frequencies of sIPSCs in *Df(16)A*^*+/−*^ neurons (0.49 ± 0.05 Hz, *n* = 15) compared to that of WT ones (0.33 ± 0.03 Hz, *n* = 16, *p* < 0.05, *t* test, Fig. 3g). At DIV14, *Df(16)A*^*+/−*^ neurons showed a comparable frequency compared to WT neurons (Fig. 3g and Table S1C). There was no significant difference in amplitude rising time or decay time of sIPSCs between genotypes at both DIV7 and DIV14 (Fig. 3g and Table S1C).

mIPSCs were further recorded in the presence of 1 µM TTX. Sample traces of mIPSCs are shown in Fig. 3f. At DIV14, the mIPSC amplitude of *Df(16)A*^*+/−*^ neurons (20.97 ± 1.28 pA, *n* = 10) was significantly increased compared to WT neurons (16.48 ± 1.28 pA, *n* = 8, *t* test, *p* < 0.05), but it was comparable at DIV7 (Fig. 3h and Table S1D). No significant changes were observed in mIPSC rising time or decay time at either DIV7 or DIV14 (Fig. 3h and Table S1D). Taken together, our data provided evidence for an increased inhibitory synaptic activity in cultured *Df(16)A*^*+/−*^ neurons.

### *Df(16)A*^*+/−*^ embryonic cortical neurons show altered calcium homeostasis

**Fig. 4:**
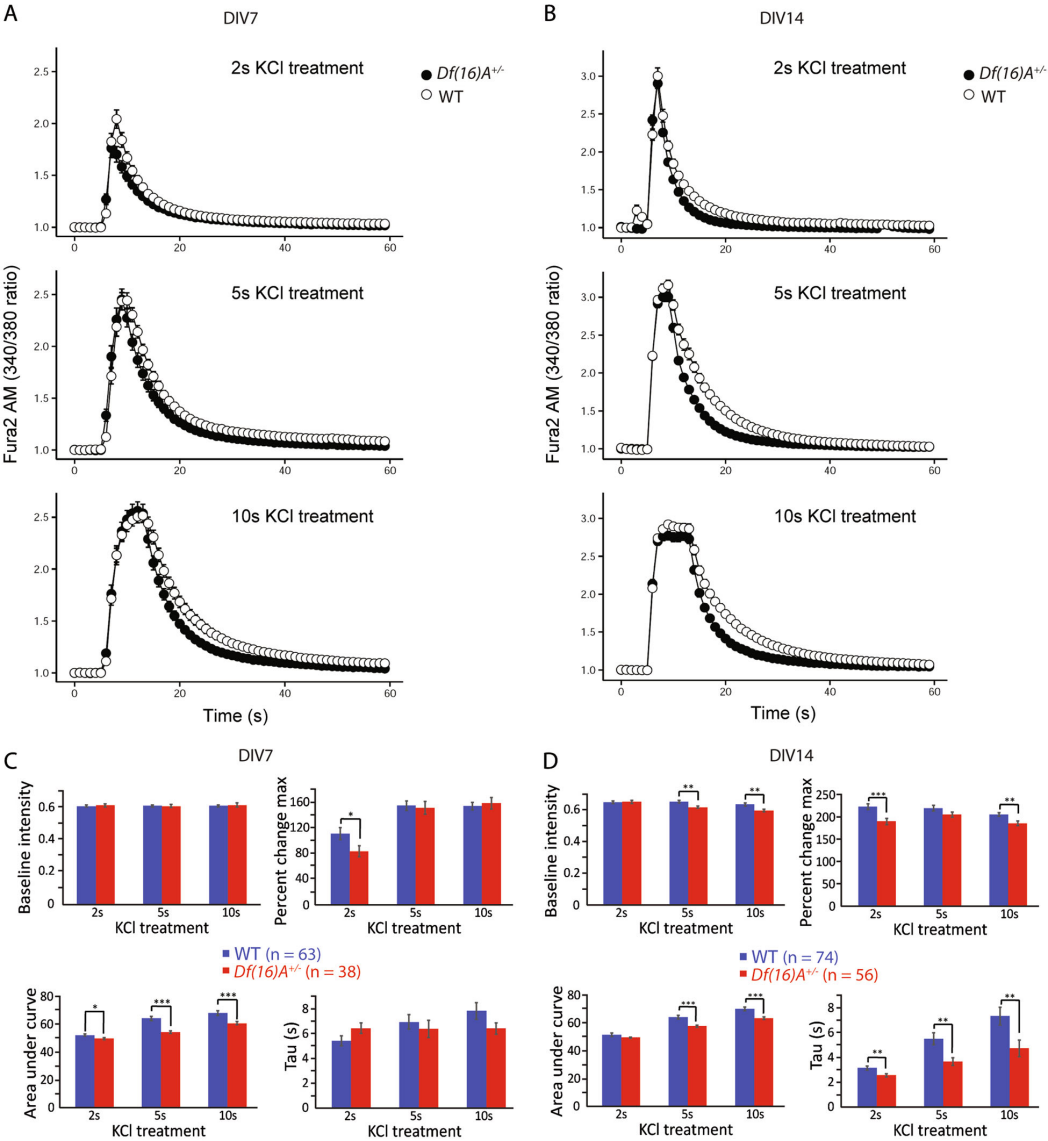
Calcium imaging of WT and *Df(16)A*^*+/−*^ cortical neurons. **a** Graph shows voltage-gated calcium responses in WT (open circles) and *Df(16)A*^*+/−*^ (closed circles) cortical neurons at DIV7 by exposing to 30 mM KCl for 2 s (top panel), 5 s (middle panel), and 10 s (bottom panel). **b** Graph shows voltage-gated calcium responses in WT (open circles) and *Df(16)A*^*+/−*^ (closed circles) cortical neurons at DIV14 by exposing to 30 mM KCl for 2 s (top panel), 5 s (middle panel), and 10 s (bottom panel). Neurons were loaded with Fura-2. Data were plotted as the ratio of fluorescence intensity excited at 340 and 380 nm vs. time and the values were normalized to the averaged baseline level. **c** Summary data of calcium imaging for WT and *Df(16)A*^*+/−*^ cortical neurons at DIV7. Compared to WT neurons, *Df(16)A*^+/−^ neuros showed significantly lower peak value of calcium elevation at 2 s and smaller AUC under all treatments. **d** Summary data of calcium imaging for WT and *Df(16)A*^*+/−*^ cortical neurons at DIV14. Significantly decreased basal calcium level, lower peak values, smaller AUC, and faster recovery were observed for *Df(16)A*^*+/−*^ neurons compared to WT ones

To probe for differences in intracellular [Ca^2+^] ([Ca^2+^]_i_) handling between WT and *Df(16)A*^*+/−*^ neurons, we analyzed KCl-evoked calcium influx using Fura-2 by exposing the same set of neurons to 30 mM KCl for 2, 5, and 10 s, respectively. Results were expressed as the ratio of fluorescence intensity excited at 340 and 380 nm vs. time and the values were normalized to the averaged baseline level (Fig. 4a,b). Basal cytosolic free calcium level was recorded following 6 s of perfusion with Ringer’s solution. Quick calcium influx was recorded upon exposure to KCl (2, 5, and 10 s) as a sharp responsive peak caused by the opening of voltage-dependent calcium channels. The elevated calcium gradually returned to the basal level upon perfusion back to Ringer’s solution. Higher evoked peaks were detected upon increasing exposure time and the response reached a plateau at 10 s, indicating a saturation of open calcium channels (Fig. 4a,b). The same set of experiments were performed at DIV14 and a pattern of similar but higher-intensity responses were obtained (Fig. 4b) compared to that at DIV7 (Fig. 4a).

We analyzed baseline intensity, peak amplitudes (percent change max), area under the curve (AUC, an index of the overall cytosolic Ca^2+^ load) and recovery phase time (*τ*) of [Ca^2+^]_i_ changes during depolarization and the ensuing recovery. Quantified data obtained at DIV7 and DIV14 are shown in Table S2 and S3, respectively. At DIV7, compared to WT neurons, *Df(16)A*^*+/−*^ neurons showed significant decrease in AUC at all three exposure durations and a significant decreased peak value at 2 s exposure only (Fig. 4c and Table S2). In contrast at DIV14, *Df(16)A*^*+/−*^ neurons exhibited more pronounced deficits in calcium signaling, including decreased baseline [Ca^2+^]_i_ levels at 5 and 10 s exposures, decreased peak values at 2 and 10 s exposure, decreased AUC at 5 and 10 s exposures, and overall faster recovery (Fig. 4d and Table S3). The calcium influx following depolarization reflects the availability of open calcium channels at cell surface and also calcium-induced calcium release from intracellular stores, while the kinetics of recovery reflect the efficiency of clearance of intracellular calcium. These results indicated that the voltage-gated calcium channels and the clearance of cytosolic calcium are altered in *Df(16)A*^*+/−*^ neurons.

### *Df(16)A*^*+/−*^ embryonic cortical neurons show altered expression of genes related to neuronal activity

**Fig. 5:**
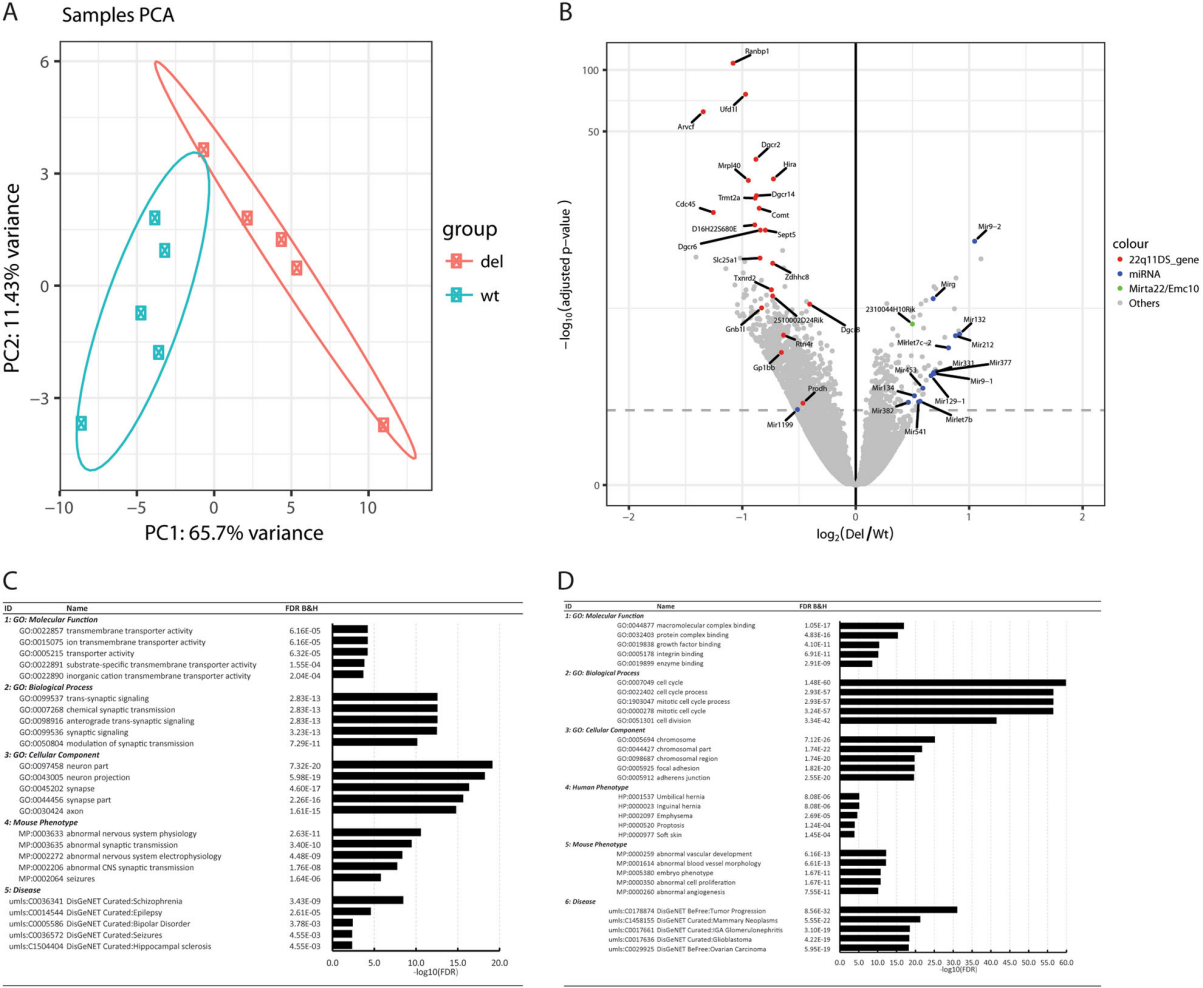
Gene expression profile analysis of WT and *Df(16)A*^*+/−*^ cortical neurons. **a** PCA analysis indicates a good separation of the expression profiles between WT (green) and *Df(16)A*^*+/−*^ (red) cortical neurons at DIV7. **b** Volcano plot indicating downregulation of the genes within the 22q11.2 deletion region and upregulation of primary miRNA genes and *Mirta22/Emc10* as previous reported. **c** GO and phenotype analysis of significantly upregulated genes (FDR adjusted *p* < 0.05). **d** GO and phenotype analysis of significantly downregulated genes (FDR adjusted *p* < 0.05)

Transcription profiles from five pairs of mutant and WT neurons at DIV7, when the majority of physiological changes are observed, were resolved using next-generation RNA sequencing technology at single-gene resolution. Principal component analysis (PCA) of the normalized counts revealed that the first principal component was strongly correlated with genotypes (64% of variance explained, Fig. 5a). The second principal component explained less variance (13%) (Fig. 5a). A total of 1350 DEGs were identified as associated with “disease” phenotype with 242 genes upregulated and 1108 genes downregulated (at FDR adjusted *p* < 0.05) in *Df(16)A*^*+/−*^ cortical neurons (Fig. 5b and Table S4). Examination of RNA-seq reads mapped to the 22q11.2 deletion region indicated that, as expected, the expression of most of genes was downregulated in mutant neurons. Importantly, we also obtained clear evidence of alterations of miRNA genes as well as of upregulation of the major target of miRNA dysregulation (*Mirta22/Emc10*^12^^[, 18^) as described previously in vivo, suggesting that cultured neurons recapitulate key transcriptional changes occurring in vivo (Fig. 5b).

To determine key functional effects, we conducted GO analysis using Toppgene^25^. This analysis revealed that upregulated genes in mutant neurons were enriched in GO terms related to “transmembrane transporter activity (GO:0022857)”, “trans-synaptic signaling (GO:0099537)”, and “neuron part (GO:0097458)” (Fig. 5c), while downregulated genes were enriched in GO terms related to “macromolecular complex binding (GO:0044877)”, “cell cycle (GO:0007049)”, and “chromosome (GO:0005694)” (Fig. 5d). These results suggest that transcriptional changes could account, at least in part, for the alterations in the physiological properties of *Df(16)A*^*+/−*^ neurons described above. To further explore the mechanistic connections between transcriptome and electrophysiological features, we looked for common functional pathways among altered genes using the generally applicable gene-set enrichment for pathway analysis (GAGE), which maps the gene expression pattern onto the Kyoto Encyclopedia of Genes and Genomes Pathway database (KEGG)^26^. GAGE indicated an enrichment of synapse related pathways among the upregulated expressed genes including “GABAergic synapse” (mmu04727), “Serotonergic synapse” (mmu04726), “Glutamatergic synapse” (mmu04724), and “Cholinergic synapse” (mmu04725), while downregulated genes showed enrichment for pathways related to DNA repair such as “Mismatch repair” (mmu03430) and “Nucleotide excision repair” (mmu03420) (Table S5). Notably, many enriched pathways contain both upregulated and downregulated genes. To obtain some initial general insights into the mechanisms underlying the electrophysiological features described above, we specifically looked into KEGG pathways related to “Glutamatergic synapse” (mmu04724), “GABAergic synapse” (mmu04727) and “Calcium signaling pathway” (mmu04020). We found that many key receptors located at glutamatergic synapses are upregulated at various levels in DIV7 *Df(16)A*^*+/−*^ neurons (Fig. S2). The most consistent changes among individual mice include nodes for AMPA receptors, NMDA receptors and kainate receptors, although only the AMPA receptor *Gria1* showed a statistically significant upregulation. Whether these alterations represent primary deficits or secondary compensations remains to be determined. We observed a similar upregulation of receptors related to GABAergic synapse pathways such as *Gabbr1* (FDR adjusted *p* = 0.01, fold change = 1.13) and *Gabbr2* (FDR adjusted *p* = 0.077, fold change = 1.15) (Table S4 and Fig. S3). These observations are overall consistent with our electrophysiological findings of increased inhibitory synaptic activity. In contrast, the calcium-signaling pathway included both up and downregulated genes (Fig. S4). Major upregulated nodes include voltage-operated calcium channels. The upregulation signals are mainly from the N-type voltage-gated calcium channels *Cacna2d2* and *Cacna1b*. In contrast, major downregulated nodes include receptors for various growth factors and effectors of G protein-coupled receptors (GPCRs). The downregulation signals are mainly from expression changes in a large number of receptors for various growth factors including connective tissue growth factor (*Ctgf*), insulin-like growth factor binding proteins (*Igfbps*), platelet-derived growth factor receptors (*Pdgfr*), fibroblast growth factor receptors (*Fgfr*), hepatoma-derived growth factor (*Hdgf*), and heparin-binding EGF-like growth factor (*Hbegf*).

## Discussion

The purpose of this study is to characterize in detail the impact of the 22q11.2 deletion on the development and function of primary embryonic cortical neurons. This information is important to facilitate mechanistic studies to determine the factors that modulate the expressivity and penetrance of this genetic lesion during neuronal development and maturation, independently of network level influences and development compensation in the intact organism. A great advantage of using cultured neurons is also that they offer a platform for the development of pharmacological screens, which will enhance the drug discovery efforts.

We investigated the physiological properties of cultured embryonic cortical neurons from mutant *Df(16)A*^*+/−*^ mice and WT mice at two different time points (DIV7 and DIV14). First, electrophysiological techniques were used to explore how the deletion affects the membrane properties, firing patterns and synaptic activity during neural development and maturation in vitro. We provide evidence that a mutation in mouse resembling a SCZ risk allele results in alterations in a number of properties of cultured cortical neurons, indicative of delayed maturation, altered synaptic activity as well as in hyper excitability of mutant neurons. Second, the perturbation of intracellular calcium homeostasis caused by the deletion was evaluated by calcium imaging under basal and KCl-depolarization conditions. Ca^2+^ signaling dysregulation has been implicated in SCZ-related diseases for a long time. Our results of Fura-2 experiments strongly suggested that deletion neurons had defects in calcium signaling, especially at DIV14, which showed significant reduction in peak amplitudes, AUC, recovery phase time and baseline level. Finally, we extracted total mRNA of cultured cortical neurons from WT and *Df(16)A*^*+/−*^ mice at DIV7 and compared their mRNA expression profiles. We found that the *Df(16)A*^*+/−*^ embryonic cortical neurons showed a predominant upregulation of ion channel-related genes, predominant downregulation of receptor-related genes and a mixed upregulation and downregulation of transporter encoding genes. Collectively, our findings suggest that transcriptional alterations of ion channels, receptors and transporters emerging as a result of the 22q11.2 deletion may ultimately affect the membrane properties, cell excitability, and calcium homeostasis of mutant neurons.

Our studies revealed three key alterations in intrinsic properties of *Df(16)A*^*+/−*^ neurons: alterations in input resistance, cell excitability, and calcium homeostasis. First, input resistance depends on the density of resting ion channels and the size of the neurons. Since the cell size (Fig. S1B) are comparable between genotypes, a higher input resistance observed in *Df(16)A*^*+/−*^ neurons at DIV7 implies fewer open channels (lower conductance). Regarding the higher *R*_in_ observed in *Df(16)A*^*+/−*^ neurons at DIV7, HCN and potassium leak channels are main contributors of *R*_in_^27–29^. Consistent with our electrophysiological results, no significant difference of HCN mRNA expression were observed between genotypes. mRNAs encoding candidate potassium leak channel isoforms in *Df(16)A*^*+/−*^ neurons showed both upregulation and downregulation compared to WTs (Table S6). *Kcnk1* and *Kcnk2*, two most abundant isoforms, showed a barely significant reduced expression, while *Kcnk3* and *Kcnk9* showed a statistically significant increase in their levels. Thus, the higher *R*_in_ observed in mutant neurons could be a combined result of transcriptional dysregulation of all these potassium leak channels. Second, since cell excitability is affected by passive membrane properties (*R*_in_ and *C*_p_), as well as voltage-dependent conductance, we investigated whether there were expression differences in voltage-gated sodium and potassium channels between genotypes that might contribute to the increased excitability in *Df(16)A*^*+/−*^ neurons (Table S7). We found that there is significant upregulation of *Scn2b* and *Scn8a* in *Df(16)A*^*+/−*^ neurons compared to WT ones. *Scn8a*, especially, encodes Nav1.6, one of the major voltage-gated sodium channels that regulate the initiation and propagation of APs in the nervous system^30,31^. Previous studies showed that loss of Nav1.6 activity results in reduced neuronal excitability, while gain-of-function mutations can increase neuronal excitability^32^. In addition, delayed rectifier K^+^-channels is another important factor regulating cell firing rate. We found that *Kcnc2*, which is essential for the generation of APs in neurons at conditions of high frequency firing^33–35^, is significantly upregulated in *Df(16)A*^*+/−*^ neurons. These findings are consistent with the observed increased AP firing rate in mutant neurons. Third, a significant decrease in AUC was detected in *Df(16)A*^*+/−*^ neurons at all three exposure durations to KCl at DIV7. We found that many ATPase-related genes were significantly upregulated in *Df(16)A*^*+/−*^ neurons (Table S8). *Atp2b2*, in particular, which encodes the plasma membrane calcium-transporter and provides an important “off” mechanism for the control of cytosolic calcium levels^36,37^, was significantly upregulated in *Df(16)A*^*+/−*^ neurons compared to WT neurons. Plasma membrane-located calcium transporter proteins clear Ca^2+^ from the cytosol against its inward gradient using energy derived from ATP hydrolysis. Therefore, increased *Atp2b2* mRNA level are consistent with our findings of decreased calcium elevation and quicker recovery rate of mutant cells. Taken together, our mRNA sequencing studies provide insights into the link between mRNA dysregulation and altered intrinsic properties of *Df(16)A*^*+/−*^ neurons, although due to the complexities of gene regulation and potential compensatory switches, there is an inherent difficulty in associating molecular changes with functional ones and distinguishing primary from secondary (compensatory) transcriptional effects. As such, potential associations between functional and molecular changes, remain to be experimentally validated.

Finally, in addition to alterations in intrinsic properties of *Df(16)A*^*+/−*^ neurons, we observed altered synaptic activity, specifically in inhibitory synaptic currents. Regarding the observed genotypic difference in mIPSCs, our list of DEGs showed some subtle but significant alterations in GABA_B_ receptors, and whether transcriptional alterations could account for the increased number of miniature inhibitory postsynaptic potentials remains to be determined. Notably, unlike mIPSCs, there were no statistically significant differences in the frequency and amplitude of mEPSCs between WT and *Df(16)A*^*+/−*^ neurons at both DIV7 and DIV14, although the mutant cells showed a trend of reduced frequency of mEPSCs at both time points (see Fig. 3b,d). These findings from mass cortical cultures (in which individual cells plated on astrocytes are innervated primarily by neighboring cells) contrasts previous findings from autaptic cultures of *Df(16)A*^*+/–*^ hippocampal neurons^8^ (in which a single isolated cell grown on an astrocytic micro-island innervates itself), which demonstrated a more robust reduction in the frequency of mEPSCs consistent with the reduced density of spines and glutamatergic synapses in mutant neurons. Such difference may be related to the cortical vs. hippocampal origin of cultured neurons but may also be dictated by differences in the spatial and temporal connectivity of neurons, in the emergence of compensatory mechanisms or both and have been previously observed in other mouse mutant lines^38^. Findings from our mass cortical cultures of embryonic neurons also differ from results obtained from recordings of *Df(16)A*^*+/−*^ adult hippocampal CA1 neurons in acute brain slices^9^, which demonstrated lower (rather than higher) frequency of sIPSCs. Overall, results obtained from synaptic analysis in dissociated cultures of embryonic neurons may not mirror accurately findings obtained in slice or in vivo recordings from adult mutant mice, which is not surprising given that synapses are formed under strict developmental constraints that are only partially recapitulated in the milieu of dissociated two-dimensional cultures.

Despite these limitations, we consider it very likely that at least some of the alterations observed here in dissociated cultures, primarily the ones in the intrinsic properties of neurons such as membrane properties, firing patterns and calcium homeostasis, contribute to the emergence of altered local multi-neuronal dynamics^17^ as well as impaired synchronization among brain regions^14^, two of the primary network deficits observed in vivo in adult 22q11.2 deletion mice (or similar, yet unidentified alteration during embryonic development) that, in turn, contribute to the cognitive and behavioral deficits reported in this mutant strain^7^. However, which one of the alterations observed in cultured neurons relate to the mouse and, eventually, the human psychiatric phenotype, what aspects of the neural substrates and, importantly, what developmental periods underlying the mouse and the human phenotypes are most faithfully modeled in neuronal cultures remain to be determined. Parallel recordings from dissociated cultures and acute brain slices from different developmental time points, accompanied by drug-based reversal of observed phenotypes in vitro and in vivo will be essential toward this end.

Comparison to human phenotype are particularly challenging due to the scarcity of brain tissue from individuals with 22q11.2 deletions (with or without comorbid psychiatric disorders) as well as the limited number of studies of patient iPSC-derived neurons from 22q11.2 deletion carriers. For example, two previous studies investigated the transcription profiles of iPSC-derived neurospheres^39^ or blood samples^40^ from 22q11.2 deletion carriers. Toyoshima et al.^39^ identified 263 upregulated and 123 downregulated genes (≥twofold change, *p* < 0.05) in patient vs. control-derived neurospheres^39^. Comparison with the top 15 altered genes listed in Table S4 revealed 4 downregulated genes in common with our study. There was no overlap between upregulated genes (Table S4) although upregulated genes in both studies are enriched for terms such as neurotransmitter receptor binding, transmission across chemical synapses and NMDA receptor activation. Comparison are complicated by the fact the samples used in the Toyoshima et al.^39^ study represent a mixed cell population of both GFAP and MAP positive cells and were not matched in reference to ethnicity and sex, as opposed to the nearly pure neurons from inbred mice in our study. Jalbrzikowski et al.^40^ identified 245 downregulated and 155 upregulated genes in blood samples of 22q11.2 deletion carriers (listed in Table S3 of Jalbrzikowski et al.^40^). Comparison with the top 15 altered genes listed in Table S4 revealed two downregulated genes in common with our study but no overlap between upregulated genes. In addition, there was little congruence between the gene networks identified as affected in these two studies. This result may not be surprising given the different cell types tested and the expectation that profiles in similar cell/tissue types might be more relevant in reflecting disease pathophysiology. Notwithstanding the evidence for variability in mRNA expression levels among studies, the most consistent and robust transcriptional dysregulation emerging due to 22q11.2 deletions, and the hemizygous deletion of the *Dgcr8* gene, is alterations in the levels of microRNA precursors and mature forms as we have previously reported in the brains of mutant mice^7^. This finding has been widely replicated in many studies including the aforementioned ones. Although several alterations are consistently observed among studies (such as changes in mir9-2, mir9-1, *Mirg*, mir132) and are also consistent with our previous report^7^, other changes are study-specific, likely due to differences in the cell types and developmental stages analyzed as well as due to methodological differences.

In summary, we used a mouse model of the SCZ-predisposing 22q11.2 microdeletion to evaluate how this genetic lesion affects cultured cortical neurons properties at the cellular, synaptic, and molecular levels. Overall, our observation of altered electrophysiological properties and calcium homeostasis as well as transcriptional profiles of cortical neurons from a 22q11.2 deletion mouse model during maturation in vitro has the potential to provide valuable insights towards revealing disease mechanisms and provide an array of potential targets that can be evaluated in downstream translational and drug-development efforts.

## Electronic supplementary material

Supplementary figure legends and table title

Figure S1

Figure S2

Figure S3

Figure S4

Table S1

Table S2

Table S3

Table S4

Table S5

Table S6

Table S7

Table S8
