# Supplementary material for: Altered function and maturation of primary cortical neurons from a 22q11.2 deletion mouse model of schizophrenia
Source: Transl Psychiatry. 2018 Apr 18;8:85. doi: 10.1038/s41398-018-0132-8 (PMC5904157; doi:10.1038/s41398-018-0132-8)
Supplement: Supplementary file 1 — Supplementary figure legends and table title [file 41398_2018_132_MOESM1_ESM.docx]

**Supplementary Figure Legends**

**Figure S1: Rheobase, transient inward and sustained outward currents of WT and Df(16**)***A^+/-^* cortical neurons.**

(A) Immunostaining of WT pyramidal cells (upper panel: EMX1 (green) and Tuj1 (red); lower panel: MAP2 (green) and Neu N (red). Scale bar = 30 µm. (B) Quantification of cell body size in WT and *Df(16)A^+/-^* cortical neurons at DIV7 and DIV14, respectively. There was no significant difference between genotypes at both time points. (C) Quantification of rheobase in WT and *Df(16)A^+/-^* cortical neurons at DIV7 and DIV14, respectively. There was no significant difference between genotypes at both time points. (D) Quantification of Tau in WT and *Df(16)A^+/-^* cortical neurons at DIV7 and DIV14, respectively. There was no significant difference between genotypes at both time points. (E) Sample traces of transient inward currents and sustained outward currents recorded from WT neurons at DIV14. Currents were induced by a series of depolarizing voltage steps from -60 mV to +60 mV with increment of 10 mV. (F) I-V curve of transient inward currents from WT (black trace) and *Df(16)A^+/-^* (red trace) cortical neurons at DIV7 and DIV14, respectively. No difference was observed between genotypes at both time points (2-way repeated measures ANOVA, genotype x current: p>0.05). (G) I-V curve of sustained outward currents from WT (black trace) and *Df(16)A^+/-^* (red trace) cortical neurons at DIV7 and DIV14, respectively. No difference was observed between genotypes at both time points (2-way repeated measures ANOVA, genotype x current: p>0.05).

**Figure S2: Differential expression patterns of Glutamatergic synapse pathway genes in *Df(16)A^+/-^* neurons at DIV7.**

Each rectangle represents a KEGG gene node (an entry point for a set of related genes. Individual altered genes are listed in Supplementary Table S4). There are five bars inside the rectangle with colors representing the fold differences between 5 pairs of mutant and WT neurons. Red indicates up-regulation; green indicates down-regulation; grey indicates no change and white indicates no data.

**Figure S3: Differential expression patterns of GABAergic synapse pathway genes in *Df(16)A^+/-^* neurons at DIV7.**

Each rectangle represents a KEGG gene node (an entry point for a set of related genes. Individual altered genes are listed in Supplementary Table S4). There are five bars inside the rectangle with colors representing the fold differences between 5 pairs of mutant and WT neurons. Red indicates up-regulation; green indicates down-regulation; grey indicates no change and white indicates no data.

**Figure S4: Differential expression patterns of calcium signaling pathway genes in *Df(16)A^+/-^* neurons at DIV7.**

Each rectangle represents a KEGG gene node (an entry point for a set of related genes. Individual altered genes are listed in Supplementary Table S4). There are five bars inside the rectangle with colors representing the fold differences between 5 pairs of mutant and WT neurons. Red indicates up-regulation; green indicates down-regulation; grey indicates no change and white indicates no data.

**Supplementary Table S1. Quantification data of synaptic activities.**

**Supplementary Table S2. Quantification data of calcium imaging at DIV7.**

**Supplementary Table S3. Quantification data of calcium imaging at DIV14.**

**Supplementary Table S4. Differential expression profile of *Df(16)A^+/-^* cortical neurons.**

**Supplementary Table S5. Altered signaling pathways detected by GAGE analysis.**

**Supplementary Table S6. Expression profile of potassium leak channels, two pore domain subfamily K (KCNK), in *Df(16)A^+/-^* cortical neurons.**

**Supplementary Table S7. Expression profile of voltage-gated sodium and potassium channels in *Df(16)A^+/-^* cortical neurons.**

**Supplementary Table S8. Expression profile of ATPase in *Df(16)A^+/-^* cortical neurons.**
