# Supplementary figures and images for: Altered function and maturation of primary cortical neurons from a 22q11.2 deletion mouse model of schizophrenia
Source: Transl Psychiatry. 2018 Apr 18;8:85. doi: 10.1038/s41398-018-0132-8 (PMC5904157; doi:10.1038/s41398-018-0132-8)

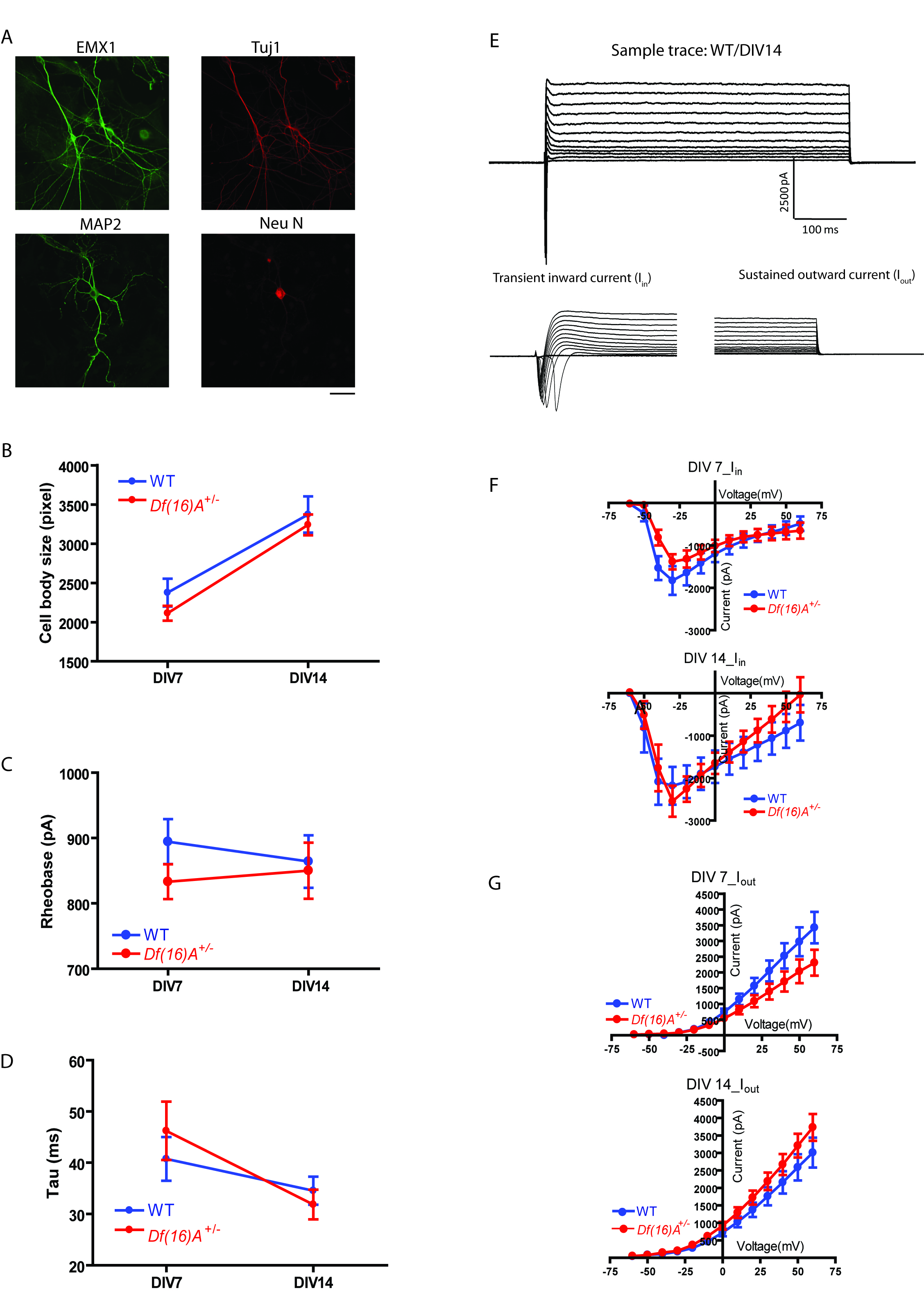

Supplement: Supplementary file 2 — Figure S1 [file 41398_2018_132_MOESM2_ESM.jpg]

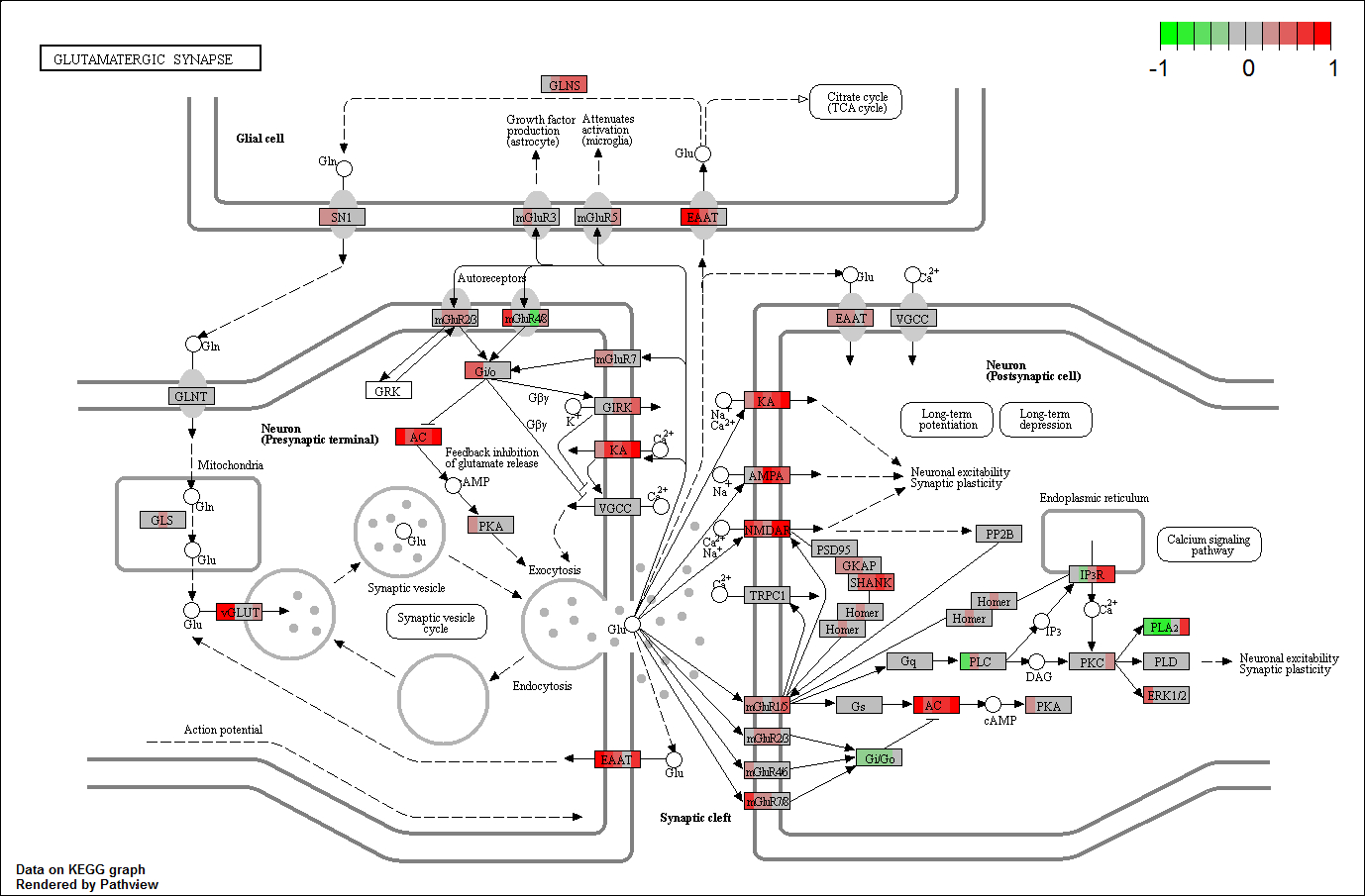

Supplement: Supplementary file 3 — Figure S2 [file 41398_2018_132_MOESM3_ESM.jpg]

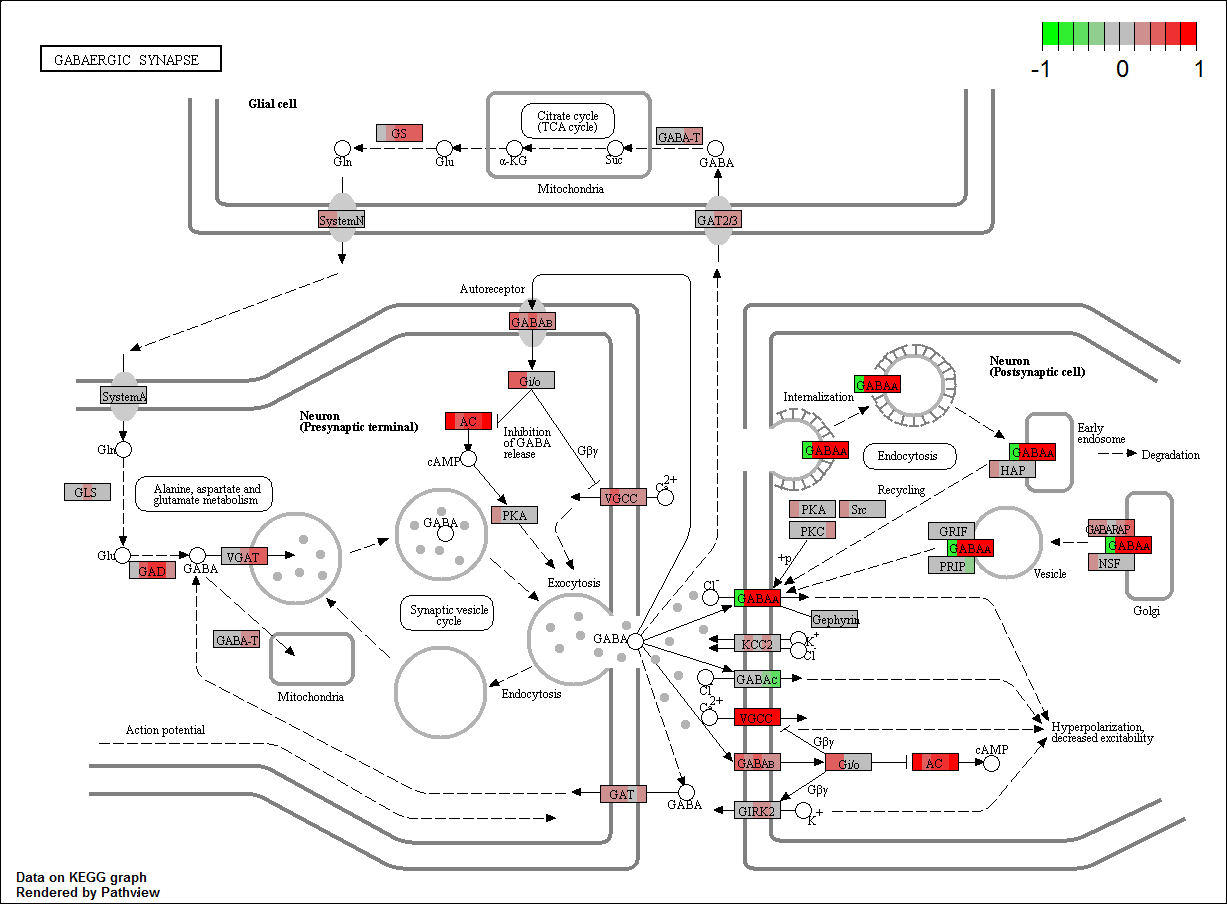

Supplement: Supplementary file 4 — Figure S3 [file 41398_2018_132_MOESM4_ESM.jpg]

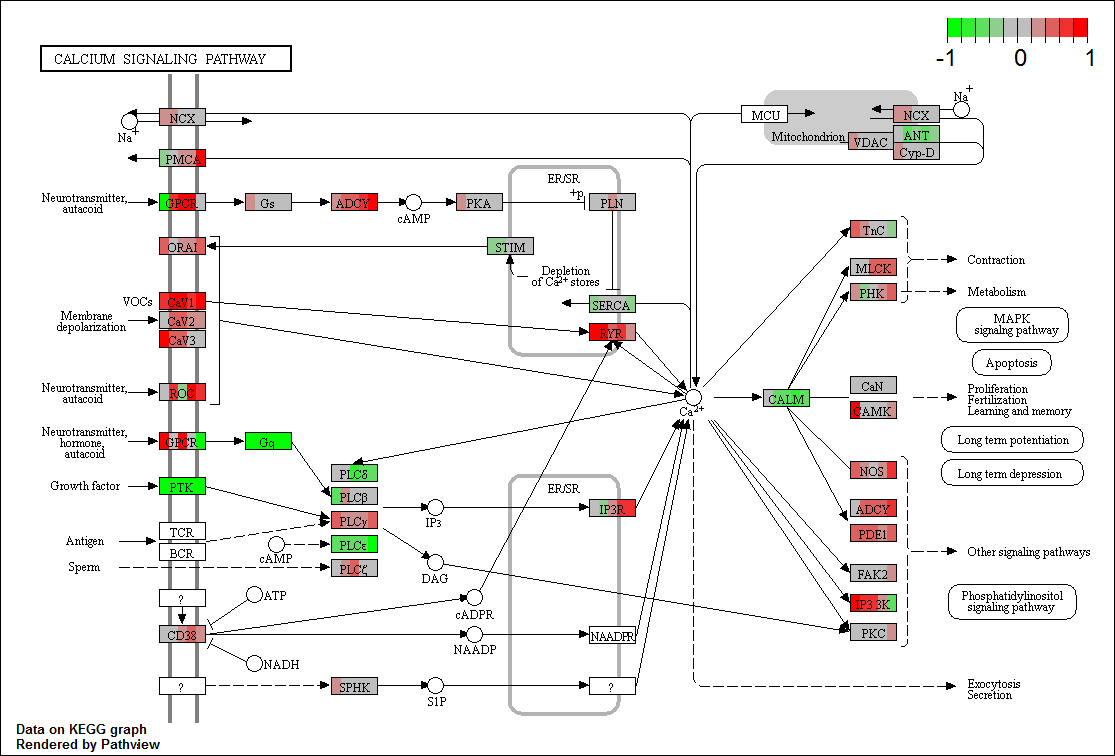

Supplement: Supplementary file 5 — Figure S4 [file 41398_2018_132_MOESM5_ESM.jpg]
