# Supplementary material for: Altered function and maturation of primary cortical neurons from a 22q11.2 deletion mouse model of schizophrenia
Source: Transl Psychiatry. 2018 Apr 18;8:85. doi: 10.1038/s41398-018-0132-8 (PMC5904157; doi:10.1038/s41398-018-0132-8)
Supplement: Supplementary file 6 — Table S1 [file 41398_2018_132_MOESM6_ESM.pdf]

Supplementary Table S1: Quantification data of synaptic activities.

Table S1A: sEPSC

Time	Genotype	Frequency	p-value	Amplitude	p-value	Rise	p-value	Decay	p-value
DIV7	WT (n = 18)	4.24 ± 0.24	0.518603	29.52 ± 1.37	0.8381	2.81 ± 0.08	0.327289	4.42 ± 0.37	0.17362
	Df(16)A+/- (n =17)	4.57 ± 0.45		29.98 ± 1.74		2.67 ± 0.11		3.83 ± 0.21	
DIV14	WT (n = 17)	10.86 ± 0.25	0.463525	33.83 ± 3.67	0.61329	2.74 ± 0.09	0.786388	5.88 ± 0.34	0.06945
	Df(16)A+/- (n =14)	11.56 ± 1.01		31.26 ± 3.28		2.78 ± 0.16		4.81 ± 0.37	

Table S1B: mEPSC

Time	Genotype	Frequency	p-value	Amplitude	p-value	Rise	p-value	Decay	p-value
DIV7	WT (n = 17)	3.32 ± 0.44	0.331769	19.99 ± 2.11	0.90242	1.91 ± 0.15	0.239713	2.46 ± 0.35	0.50384
	Df(16)A+/- (n = 19)	2.75 ± 0.37		19.61 ± 2.44		2.19 ± 017		2.76 ± 0.29	
DIV14	WT (n = 7)	9.74 ± 2.79	0.634158	19.32 ± 3.01	0.67871	2.15 ± 0.25	0.545745	2.98 ± 0.46	0.06538
	Df(16)A+/- (n = 8)	8.19 ± 1.81		17.78 ± 2.21		2.34 ± 0.19		1.96 ± 0.27	

Table S1C: sIPSC

Time	Genotype	Frequency	p-value	Amplitude	p-value	Rise	p-value	Decay	p-value
DIV7	WT (n = 16)	0.33 ± 0.03	0.021361	23.27 ± 1.95	0.67658	4.33 ± 0.36	0.546525	12.12 ± 0.63	0.24129
	Df(16)A+/- (n = 15)	0.49 ± 0.05		22.19 ± 1.64		4.65 ± 0.39		13.33 ± 0.79	
DIV14	WT (n = 17)	1.23 ± 0.18	0.085309	31.16 ± 3.41	0.58668	4.93 ± 0.49	0.951298	11.84 ± 1.78	0.49087
	Df(16)A+/- (n = 17)	1.65 ± 0.16		28.96 ± 2.12		4.97 ± 0.46		13.43 ± 1.43	

Table S1D: mIPSC

Time	Genotype	Frequency	p-value	Amplitude	p-value	Rise	p-value	Decay	p-value
DIV7	WT (n = 11)	0.29 ± 0.09	0.302667	16.61 ± 1.64	0.52422	2.09 ± 0.12	0.137232	11.84 ± 1.19	0.23063
	Df(16)A+/- (n = 12)	0.45 ± 0.12		15.42 ± 0.99		2.33 ± 0.11		13.49 ± 0.73	
DIV14	WT (n = 8)	1.08 ± 0.19	0.535187	16.48 ± 0.86	0.01377	2.02 ± 0.03	0.462422	13.04 ± 0.46	0.76004
	Df(16)A+/- (n = 10)	1.31 ± 0.28		20.97 ± 1.28		2.06 ± 0.04		12.78 ± 0.63	
