# Supplementary material for: Altered function and maturation of primary cortical neurons from a 22q11.2 deletion mouse model of schizophrenia
Source: Transl Psychiatry. 2018 Apr 18;8:85. doi: 10.1038/s41398-018-0132-8 (PMC5904157; doi:10.1038/s41398-018-0132-8)
Supplement: Supplementary file 7 — Table S2 [file 41398_2018_132_MOESM7_ESM.pdf]

Supplementary Table S2: Quantification data of calcium imaging (DIV7).

Genotype	KCl Treatment	Variable	n cells	Mean	Median	SD	SE	P value
WT	2s	Baseline_intensity	63	0.620357	0.6135	0.054671	0.006888	0.67
Df(16)A+/-			38	0.625105	0.61875	0.054758	0.008883	
WT		Percent_change_max	63	110.9841	88	72.4031	9.121934	0.03
Df(16)A+/-			38	83.13158	67.5	53.98707	8.757859	
WT		Area_Under_curve	63	52.363	51.122	6.315742	0.795709	0.027
Df(16)A+/-			38	49.95768	49.5745	4.412177	0.71575	
WT		Tau_s	63	5.44741	4.619	3.201851	0.403395	0.083
Df(16)A+/-			37	6.460703	6.5641	2.518722	0.414075	
WT	5s	Baseline_intensity	63	0.622762	0.6155	0.053279	0.006713	0.86
Df(16)A+/-			38	0.620803	0.61	0.054463	0.008835	
WT		Percent_change_max	63	155.0952	159	58.36695	7.353544	0.77
Df(16)A+/-			38	151.4211	143.5	62.08566	10.07162	
WT		Area_Under_curve	63	64.4271	63.341	11.1037	1.398934	0.00000011
Df(16)A+/-			38	54.58287	53.996	6.177638	1.002145	
WT		Tau_s	58	6.962998	5.7394	4.509553	0.592133	0.55
Df(16)A+/-			37	6.402405	5.2309	4.310545	0.708649	
WT	10s	Baseline_intensity	63	0.622405	0.6095	0.056475	0.007115	0.7
Df(16)A+/-			38	0.627842	0.608	0.074203	0.012037	
WT		Percent_change_max	63	154.4762	162	47.50683	5.985298	0.69
Df(16)A+/-			38	158.7368	171.5	54.59467	8.856424	
WT		Area_Under_curve	63	68.28151	64.685	12.97799	1.635073	0.00039
Df(16)A+/-			38	60.99766	61.488	6.917156	1.122111	
WT		Tau_s	52	7.85469	6.619	4.82183	0.668667	0.082
Df(16)A+/-			36	6.42665	5.7212	2.760673	0.460112	
