# Supplementary material for: Altered function and maturation of primary cortical neurons from a 22q11.2 deletion mouse model of schizophrenia
Source: Transl Psychiatry. 2018 Apr 18;8:85. doi: 10.1038/s41398-018-0132-8 (PMC5904157; doi:10.1038/s41398-018-0132-8)
Supplement: Supplementary file 8 — Table S3 [file 41398_2018_132_MOESM8_ESM.pdf]

Supplementary Table S3: Quantification data of calcium imaging (DIV14).

Genotype	KCl Treatment	Variable	n cells	Mean	Median	SD	SE	P value
WT	2s	Baseline_intensity	74	0.651959	0.62975	0.080011	0.009301	0.83
Df(16)A+/-			56	0.654777	0.63925	0.070695	0.009447	
WT		Percent_change_max	74	222.9865	229	56.20327	6.533498	0.00054
Df(16)A+/-			56	189.6071	184	50.54383	6.754204	
WT		Area_Under_curve	74	51.59543	53.122	11.39124	1.324205	0.16
Df(16)A+/-			56	49.57332	49.557	3.734037	0.498982	
WT		Tau_s_2	66	3.472858	3.057	1.587852	0.195451	0.007
Df(16)A+/-			52	2.81364	2.54625	1.002918	0.13908	
WT	5s	Baseline_intensity	74	0.65575	0.63275	0.07866	0.009144	0.0019
Df(16)A+/-			56	0.619821	0.6205	0.050202	0.006709	
WT		Percent_change_max	74	219.1351	208.5	54.8603	6.377381	0.1
Df(16)A+/-			56	204.7143	200.5	44.36531	5.928564	
WT		Area_Under_curve	74	64.2453	63.2795	11.11209	1.291754	0.000025
Df(16)A+/-			56	57.76089	57.233	5.313298	0.710019	
WT		Tau_s_2	66	6.090915	4.83675	4.270713	0.525688	0.0019
Df(16)A+/-			48	4.04811	3.18145	2.542661	0.367002	
WT	10s	Baseline_intensity	74	0.637716	0.6155	0.080049	0.009306	0.0017
Df(16)A+/-			47	0.599032	0.5975	0.052582	0.00767	
WT		Percent_change_max	74	205.3108	201.5	39.43919	4.584713	0.007
Df(16)A+/-			47	185.3404	182	38.49409	5.614939	
WT		Area_Under_curve	74	70.35988	67.97	11.25366	1.308211	0.000075
Df(16)A+/-			47	63.54164	62.728	7.023169	1.024435	
WT		Tau_s_2	63	8.135479	5.4109	6.402471	0.806635	0.0093
Df(16)A+/-			42	5.237076	3.74025	4.783381	0.738092	
