# Supplementary material for: Altered function and maturation of primary cortical neurons from a 22q11.2 deletion mouse model of schizophrenia
Source: Transl Psychiatry. 2018 Apr 18;8:85. doi: 10.1038/s41398-018-0132-8 (PMC5904157; doi:10.1038/s41398-018-0132-8)
Supplement: Supplementary file 11 — Table S6 [file 41398_2018_132_MOESM11_ESM.pdf]

Supplementary Table S6: Expression profile of potassium leak channels, two pore domain subfamily K (KCNK), in Df(16)A+/- cortical neurons.

GeneSymbol	baseMean	log2FoldChange	lfcSE	stat	P value	padj
Kcnk1	1010.952035	-0.468751948	0.170524104	-2.748889672	0.005979751	0.054232
Kcnk2	1426.992376	-0.276395165	0.102909831	-2.685799429	0.007235651	0.06252
Kcnk3	476.4185786	0.214075715	0.073491652	2.912925606	0.003580599	0.037541
Kcnk4	48.14588845	0.1771375	0.189506013	0.93473287	0.349925983	0.624438
Kcnk5	15.77097643	-0.709757518	0.259426535	-2.735870945	0.006221542	0.055796
Kcnk6	4.186742753	-0.351979418	0.28934272	-1.216479259	0.223802407	NA
Kcnk7	1.045604755	-0.004314639	0.226080036	-0.019084564	0.984773645	NA
Kcnk9	916.57136	0.321660129	0.077561026	4.147187656	3.37E-05	0.000943
Kcnk10	609.9467234	-0.148696939	0.103836513	-1.432029393	0.152135409	0.410082
Kcnk12	102.3069837	0.062103338	0.189123358	0.328374763	0.742628322	0.89249
Kcnk13	78.8904367	0.109694079	0.200298155	0.547653964	0.583929528	0.801754
Kcnk15	0.758133188	-0.193293118	0.200489304	-0.964106887	0.33499234	NA
Kcnk16	0	NA	NA	NA	NA	NA
Kcnk18	0.658011244	-0.283674467	0.199207377	-1.424015872	0.154441878	NA
