# Supplementary material for: Altered function and maturation of primary cortical neurons from a 22q11.2 deletion mouse model of schizophrenia
Source: Transl Psychiatry. 2018 Apr 18;8:85. doi: 10.1038/s41398-018-0132-8 (PMC5904157; doi:10.1038/s41398-018-0132-8)
Supplement: Supplementary file 12 — Table S7 [file 41398_2018_132_MOESM12_ESM.pdf]

Supplementary Table S7: Expression profile of voltage-gated sodium and potassium channels in Df(16)A+/- cortical neurons.

Sodium channels, voltage gated (SCN)

GeneSymbol	baseMean	log2FoldChange	lfcSE	stat	P value	padj
Scn1a	2839.5863	0.129366036	0.131179	0.986178	0.324046	0.599771
Scn1b	1152.5285	0.315664607	0.135027	2.337781	0.019399	0.121791
Scn2a1	4532.102	0.160934687	0.062243	2.585602	0.009721	0.077444
Scn2b	748.87013	0.446569862	0.091685	4.870685	1.11E-06	6.93E-05
Scn3a	2031.3436	0.12620778	0.079633	1.584866	0.112997	0.349069
Scn3b	4264.859	0.211112433	0.11197	1.885438	0.059371	0.241905
Scn4a	0.6512144	0.060686914	0.169408	0.358229	0.720172	NA
Scn4b	19.430738	-0.126209552	0.236981	-0.53257	0.59433	0.808461
Scn5a	122.23498	-1.312606914	0.262996	-4.99097	6.01E-07	4.26E-05
Scn7a	5.9270248	-0.183031157	0.284312	-0.64377	0.519726	NA
Scn8a	3581.5583	0.194270496	0.063256	3.07117	0.002132	0.025213
Scn9a	25.396361	0.295392991	0.231617	1.27535	0.202185	0.477705
Scn10a	0.5099092	-0.05773531	0.17809	-0.32419	0.745793	NA
Scn11a	6.6006126	0.413101327	0.283275	1.458304	0.144757	NA

Potassium channel, voltage-dependent, Delayed rectifier

GeneSymbol	baseMean	log2FoldChange	lfcSE	stat	P value	padj
Kcna1	2211.1322	0.073037524	0.204809	0.356612	0.721382	0.880049
Kcna2	1957.9713	0.256404948	0.111853	2.292333	0.021886	0.132098
Kcna3	278.866	0.056898123	0.153098	0.371646	0.710156	0.874804
Kcna5	56.965172	0.176533913	0.175853	1.003873	0.31544	0.59186
Kcna6	2185.7843	0.093480315	0.065844	1.419721	0.155689	0.415829
Kcna7	2.6051406	0.000517386	0.276858	0.001869	0.998509	NA
Kcna10	1.0711445	-0.032012019	0.225717	-0.14182	0.887219	NA
Kcnb1	1673.6636	0.100046868	0.061233	1.633876	0.102285	0.330038
Kcnb2	222.87871	-0.019377674	0.118973	-0.16287	0.870618	0.946729
Kcnc1	2136.3368	0.02129239	0.070193	0.303339	0.761632	0.900029
Kcnc2	968.51837	0.447671696	0.1482	3.020717	0.002522	0.028861
Kcnq1	4.2364629	0.077521109	0.289233	0.268023	0.788681	NA
Kcnq2	3121.4387	0.184016322	0.073583	2.500794	0.012392	0.091384
Kcnq3	303.29361	0.189940697	0.118686	1.600364	0.109518	0.342789
Kcnq4	178.47542	0.082029269	0.119621	0.685742	0.492876	0.741306
Kcnq5	3196.8921	0.057383551	0.06007	0.955273	0.339439	0.615266
Kcnh1	889.42251	0.155243982	0.102582	1.513368	0.130186	0.378029
