# Supplementary material for: Altered function and maturation of primary cortical neurons from a 22q11.2 deletion mouse model of schizophrenia
Source: Transl Psychiatry. 2018 Apr 18;8:85. doi: 10.1038/s41398-018-0132-8 (PMC5904157; doi:10.1038/s41398-018-0132-8)
Supplement: Supplementary file 13 — Table S8 [file 41398_2018_132_MOESM13_ESM.pdf]

Supplementary Table S8. Expression profile of ATPase in Df(16)A+/- cortical neurons.

GeneSymbol	baseMean	og2FoldChang	lfcSE	stat	pvalue	padj
Atp1a2	4678.399459	0.705926306	0.105470105	6.69314119	2.18E-11	1.01E-08
Atp13a4	26.74084902	0.907026638	0.174394061	5.20101792	1.98E-07	2.10E-05
Atp6v0e	464.8403415	-0.31903324	0.078146648	-4.0824943	4.46E-05	0.001354
Atp6v1e1	4804.202585	0.092187624	0.031851201	2.89432172	0.0038	0.04367
Atp10a	233.4278127	-0.35011336	0.11209071	-3.1234823	0.001787	0.021837
Atp2b2	6106.021849	0.216019607	0.069692229	3.0996226	0.001938	0.02337
Atp6v0a1	10627.23312	0.153180663	0.051482581	2.97538817	0.002926	0.03219
Atp7b	221.3225009	-0.63156702	0.219770473	-2.8737574	0.004056	0.040682
Atp6v1a	13080.98748	0.112066107	0.039880476	2.81004939	0.004953	0.04701
